# The Patello-Femoral Joint Degeneration and the Shape of the Patella in the Population Needing an Arthroscopic Procedure

**DOI:** 10.3390/medicina54020021

**Published:** 2018-04-24

**Authors:** Rimtautas Gudas, Laimonas Šiupšinskas, Agnė Gudaitė, Vladas Vansevičius, Edgaras Stankevičius, Alfredas Smailys, Akvilė Vilkytė, Rasa Simonaitytė

**Affiliations:** 1 Institute of Sports of Lithuanian University of Health Sciences, LT-44307 Kaunas, Lithuania; laimonas.siupsinskas@lsmuni.lt (L.Š.); agne.gudaite@lsmuni.lt (A.G.); akvile.vilkyte@gmail.com (A.V.); rasa.simonaityte@lsmuni.lt (R.S.); 2 Sports Trauma and Arthroscopic Unit of Lithuanian University of Health Sciences, LT-44307 Kaunas, Lithuania; alfredas.smailys@kaunoklinikos.lt; 3 Vilnius University Observatory, Ciurlionio 29, LT-03100 Vilnius, Lithuania; vladas.vansevicius@ff.vu.lt; 4 Center for Physical Sciences and Technology, Savanoriu 231, LT-02300 Vilnius, Lithuania; 5 Institute of Physiology and Pharmacology of Lithuanian University of Health Sciences, LT-44307 Kaunas, Lithuania; Edgaras.Stankevicius@lsmuni.lt

**Keywords:** articular cartilage, patella shape, Wiberg type, knee joint, physical activity

## Abstract

*Background*: the main goal of the study was to investigate the prevalence of the articular cartilage defects (ACD) in the patellofemoral (PF) region of the knee joint based on the anatomical shapes of patella and its impact on the level of physical activity in the population needing arthroscopic procedures for all types of pathologies in the knee. *Methods*: The articular cartilage status of the PF region was obtained from 1098 arthroscopic procedures of the knee joint. The ACD were correlated to Wiberg’s shape of the patella and classified according to the degree, size and depth of the ACD in the PF region using the ICRS (International Cartilage Repair Society) system: group I consisting of patients with Wiberg type I shape (W1), group II—patients with Wiberg type II shape (W2) and group III—patients with Wiberg type III shape (W3). The Tegner physical activity scale was used to evaluate the physical activity of the patients. *Results*: The mean of ACD size (PF region) in the W3 group was 3.10 ± 0.99 cm^2^, which was a statistically significantly larger area in comparison with the W1 (1.90 ± 0.63 cm^2^; *p* < 0.0000) and W2 (1.95 ± 0.71 cm^2^; *p* < 0.0000). The patients from the W3 group (mean 3.10 ± 0.99) were less physically active (<4 Tegner) compared to the W2 group (mean of 4.48 ± 0.88; *p* = 0.004) and W1 group (mean of 4.55 ± 0.72; *p* = 0.002). *Conclusions*: The patients with the Wiberg type III patella shape had a higher incidence and larger size of ACD in the PF of the knee compared to the groups of Wiberg type I and II. Wiberg III patients with a lower level of physical activity had a larger size of ACD in the PF joint.

## 1. Introduction

One-third of the elderly population is affected by osteoarthritis (OA) in their knee(s) [1], which is a disease that has a significant impact on a patient’s activities of daily living, physical activity and independence. Traditionally, knee OA has been viewed primarily as a disorder of the tibiofemoral joint (TFJ) as radiographic assessment has tended to focus only on the anteroposterior X-ray, which does not image the patellofemoral joint (PFJ). In fact, the PFJ is one of the most commonly affected compartments and is often involved in the degenerative changes of the knee [2,3,4]. Articular cartilage defects (ACD) in the PF region are more common with various dysplastic shapes of the patella [5,6,7,8]. Despite many scientific studies, the clear reason for the occurrence of articular cartilage defects (ACD) in the patellofemoral (PF) region is still poorly understood [8,9,10,11,12,13,14,15,16]. Furthermore, there is no clear correlation described between the ACD in the PF regions and the shape of the patella [9,10,11,12,16]. Very few studies have been conducted in the past to investigate the correlation between ACD in the PF region and patella shape [12,13,15,17,18]. These studies have shown variable results and generally, the data have not been found to be clinically significant. However, a small number of investigations have revealed correlations between the shape of the patella and ACD in the PF region [19,20,21]. These correlations between ACD and the shape of the patella would be extremely important for understanding the mechanisms of ACD in the PF region.

The main goal of this study was to investigate the prevalence of the articular cartilage defects (ACD) in the patellofemoral (PF) region of the knee joint based on the anatomical shapes of patella and its impact on the level of physical activity in the population needing an arthroscopic procedure.

## 2. Materials and Methods

All procedures and investigations performed in the present study were in accordance with the ethical standards of the institutional and national research committee and with the 1964 Declaration of Helsinki and its later amendments and comparable ethical standards (BE-2-22). The data related to the articular cartilage status of patellofemoral region were obtained from 1098 arthroscopic procedures of the knee joint in the period of 2005–2010. According to our research protocol, each patient prior to the arthroscopic knee procedure underwent X-ray in the Merchant view or MRI investigations in the outpatient clinic to determine the shape of the patella. Independent observers performed all the patella measurements. The investigated first involved the assessment of the Wiberg‘s type [11,12]. The Wiberg’s classification was used on a skyline radiograph at 30 degrees of flexion and consisted of three types. For Type I, the medial facet has a concave shape and has almost the same area of the lateral facet (this is present in 16.1% of the healthy population according to Servien et al.). For Type II, the medial facet still has a concave shape, but this is smaller than the lateral face (this is present in 80% of the healthy population). For Type III, the medial facet has a convex shape and is almost vertical (this is present in 12.9% of the healthy population [22]). In Wiberg type I—normal patella, with the medial and lateral facets being symmetrical and of equal size. In Wiberg type II—mild dysplasia, with the medial facet being only half the size of the lateral facet. In Wiberg type III—severe dysplasia, with the medial facet being markedly smaller and a quarter of the size of the lateral facet. A total of 406 (37%) women and 692 (63%) men with a mean age of 26 years (from 15 to 40 years) were analyzed. The case history data, arthroscopic protocols, MRI scans and X-ray radiographs, the articular cartilage defects classified based on the ICRS scores and Tegner activity scale were analyzed.

The patients were divided into 3 patella shape groups according to the MRI and/or X-ray. Group I included the patients with Wiberg type I shape (W1 group), Group II included the patients with Wiberg type II shape (W2 group) and Group III included the patients with Wiberg type III shape (W3 group). After this, the arthroscopies of the knee were performed on a regular basis for different pathologies and articular cartilage defects were inspected and classified according to the degree, size and depth of the ACD in the PF region using the International Cartilage Repair Society (ICRS) articular cartilage classification system [23]. The patients were excluded from the study if they had osteoarthritis or other systemic diseases. The Tegner activity score (TAS) was used to determine the level of physical activity of the patients [23].

### Statistical Methods

The data were stored in a Microsoft Access database. The statistical analyses were made using the Statistical Package for the Social Sciences 17 version (SPSS, Chicago, IL, USA). The sample size required was calculated based on the expected ACD size of 0.5 cm^2^, with a group size of 32 required for a power of 80% and α of 0.05. The Mann-Whitney U and Kruskal-Wallis tests were used to compare the three Wiberg types of patella between the three groups and for all variables. The results with a *p* value of ≤ 0.05 were considered to be statistically significant.

## 3. Results

**Figure 1:**
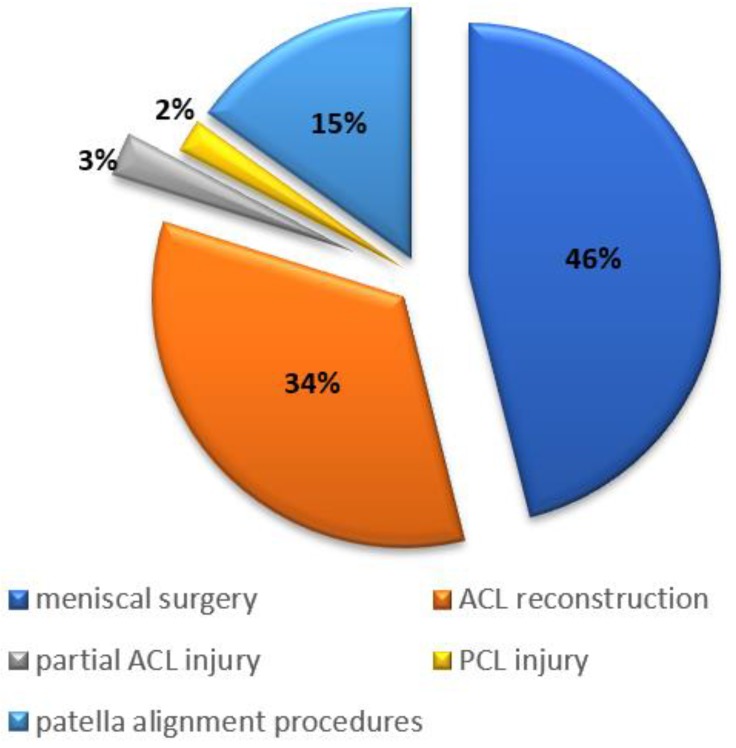
Distribution of articular cartilage degeneration and associated knee pathologies.

All arthroscopic procedures were performed due to different knee pathologies. Menisci ruptures had the highest prevalence of associated cartilage lesions in the patellofemoral region (46%), followed by anterior cruciate ligament injuries (34%) and patellar instability (15%), which is illustrated in Figure 1. The previous arthroscopic procedures had been performed in 34 of 1098 (3%) of the knees, which was most commonly due to the meniscal lesions.

Out of 1098 arthroscopic knee surgeries, articular cartilage defects (ICRS grade 1–4) were detected in 779 (71%) knees. Out of 1098 patients, an acute onset of the knee symptoms was reported in 43% and a gradual onset in 57%. Grade 3–4 (ICRS) articular cartilage defects were most commonly located in the patellofemoral (33%) and lateral femoral condyle (34%) regions, followed by the lateral tibial (18%) and medial femoral condyles (2%). According to our findings, articular cartilage defects in the patellofemoral region were detected in 343 (44%) patients. The distribution of ACD in the PF region according to the patella shape was analyzed: 9.6% (N = 33) of the defects were detected in Wiberg type I group, 58.6% (n = 201) in Wiberg type II and 31.7% (n = 109) in Wiberg type III group.

A total of 144 (42%) ACD in the patellofemoral region were larger than 2 cm^2^, which constituted 19% of all knees (Table 1). A full-thickness ACD (ICRS 3–4) in the PF region with a square area of more than 2 cm^2^ was observed in 49 (5%) knees (Table 1). Superficial ACD defects (ICRS 1–2) were significantly smaller than 2 cm^2^ (*p* = 0.001), compared to other ACDs that were larger than 2 cm^2^ (ICRS 3–4; *p* = 0.02; Table 1).

**Figure 2:**
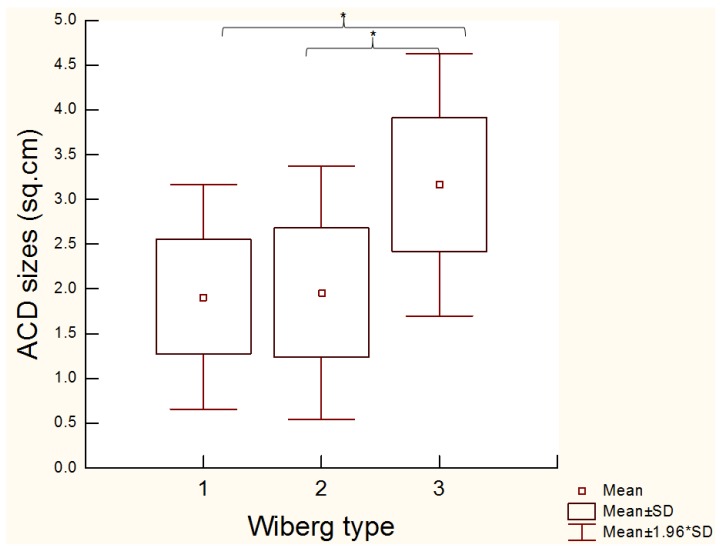
The size of articular cartilage degeneration (ACD) compared to the Wiberg’s patella shape I, II and III.

The most severe ACD cases were documented in patients with the Wiberg type III patella shape. In these patients, the mean of ACD size was 3.16 ± 0.74 cm^2^, which was statistically significantly larger area when compared to the Wiberg type I (1.90 ± 0.63 cm^2^; *p* < 0.0000) and Wiberg type II (1.95 ± 0.71 cm^2^; *p* < 0.0000; Figure 2).

The most severe ACD sizes were documented in the patients with Wiberg type III patella shapes. The Wiberg type III shapes had a significant larger ACD compared to the Wiberg type II (*p* < 0.0000) and Wiberg I shapes (*p* < 0.0000).

**Figure 3:**
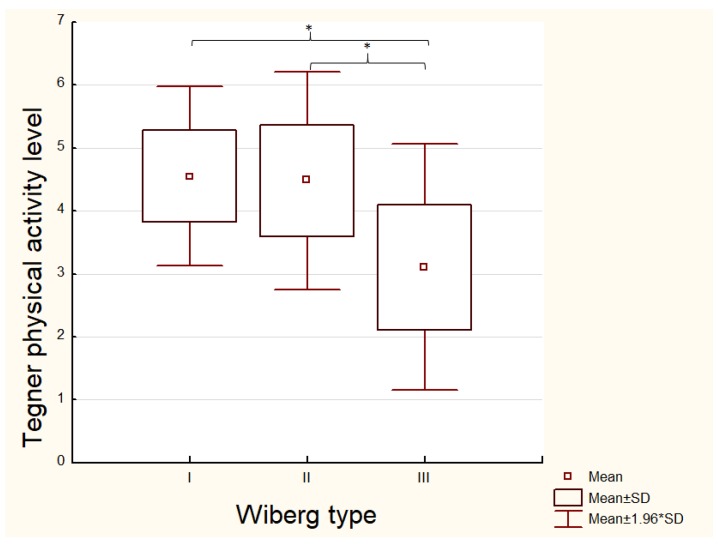
Patient’s physical activity level according to the Wiberg type of the patella shape. Wiberg type I and Wiberg type II activity levels was compared to Wiberg type III group *.

The association between the ACD size and the level of physical activity was analyzed. The cut-off point of the Tegner activity level scale was chosen to be 4, which means that the patients were able to perform moderately heavy labor. The larger articular cartilage defects in the PF region were detected in patients with lower Tegner physical activity levels (<4 Tegner). A smaller ACD (<2 cm^2^) were detected in more physically active (>4 Tegner) patients (*p* = 0.03). Patients with a larger ACD size (>2 cm^2^) had a significantly lower level of physical activity compared with those having <2 cm^2^ ACD lesions (*p* = 0.04; Table 2). The shape of the patella had the influence on the level of physical activity. We found that patients with a Wiberg type III patella shape (mean of 3.10 ± 0.99) were less physically active (<4 Tegner) compared to Wiberg type II (mean of 4.48 ± 0.88; *p* = 0.004) and Wiberg type I (mean of 4.55 ± 0.72; *p* = 0.002). The patients with Wiberg type I and II patella shapes had a similar level of physical activity (*p* = 0.51; Figure 3).

## 4. Discussion

Our study suggests that a significantly higher number of ACD cases in the PF region were documented in patients having a patella with a Wiberg type III shape. Furthermore, the ACD occurring in the PF region have an impact on the level of physical activity in the population needing an arthroscopic procedure. We found no significant difference in the distribution of ACD between Wiberg type I and II in our study. Wiberg himself stated that type I and II shapes of patella are less vulnerable to ACD in the PF region. Unfortunately, he has no substantiated findings showing an association between ACD and different shapes of the patella [11]. Furthermore, Wiberg showed that the shape of the patella was correlated with anterior knee pain and that the type III anatomical configuration of the patella leads to ACD, while the type I could be emphasized as ideal [11]. This theory was not widely accepted and most subsequent studies had shown no correlations between the shape of patella and ACD in the PF regions [1,7,17]. Potentially, the main reason for this negative correlation could be due to the small number of the investigated cases. As a result, no recent studies have been published to support Wiberg’s theory.

According to our data, the articular cartilage defects in the patellofemoral region were detected in 44% of patients and confirmed the theory that the patellofemoral region is the one of the most pregnable locations for ACD of the knee joint. The importance of the patella shape to the articular cartilage degeneration has been also widely discussed in the literature [7,14,24]. Based on the anatomical studies, Wiberg type II patella was found most frequently (59%), followed by Wiberg type I (24%) and Wiberg type III (19%) [14]. In our study, Wiberg type I consisted 12% of the group, Wiberg type II consisted 60% and Wiberg type III consisted 28%. However, other authors have noted no association between Wiberg type III and ACD in the PF region [8,12,15]. Concurrent studies have also stated that there is a significant association between Wiberg type III shape and the grade of trochlear dysplasia [3,8].

In our study, we observed that patients with Wiberg type III shape of the patella have lower levels of physical activity compared to patients with Wiberg type I and II shapes. Wiberg type I and II patients had the same level of physical activity. This suggests that the shape of the patella could be a principal anatomical factor influencing the patellofemoral biomechanics of the knee joint and the incidence of ACD. In a study by Lancourt et al., a correlation was found between the Wiberg’s classification of the patella and the width of the lateral patellofemoral ligament. The authors showed that the more that a patella shape resembled Wiberg type II, the broader the lateral patellofemoral ligament was [14]. They concluded that the properties of the patellofemoral ligament may indirectly predispose to patellofemoral instability, which has been shown to have an influence on the formation of ACD in this particular region.

A lower level of physical activity in our patients was associated with a larger size of ACD. However, it is unclear if the size of ACD itself or the shape of the patella was the reason for the decreased level of physical activity in these patients. This implies that there is a correlation between the anatomical shape of the patella and the size of ACD in the PF region. Patients with Wiberg type III patella and ACD in the PF region tend to be less physically active. Otherwise, the negative findings in the clinical tests of patellar pain and crepitation, the lack of bilateral symptoms during the follow-up, low body height and young age predict good long-term outcomes of chronic patellofemoral pain syndrome [25].

The main limitation of our study was the lack of other subjective and objective scoring systems for PF pain and their relations to the shape of the patella and articular cartilage degeneration in the patellofemoral region. Further studies need to be performed to look at the correlations between the shape of patella, ACD and clinical outcomes.

## 5. Conclusions

The patients with the Wiberg type III patella shape had a higher incidence and larger size of articular cartilage defects in the patellofemoral joint of the knee compared to the groups of Wiberg type I and II. The lower level of physical activity of the Wiberg III patients was associated with a larger size of the articular cartilage defects in the patellofemoral joint. This suggests that the Wiberg classification of anatomical shape of the patella obtained from radiological data may be a good clinical predictor of the risk of articular cartilage defects and the knee-related physical activity level of the patients. Based on our findings, we concluded that the anatomical shape of the patella is an important anatomic parameter, which may reflect the development of articular cartilage defects in the patellofemoral region.

## Figures and Tables

**Table 1:** Superficial and deep ACD defects according to size.

ACD Size	ICRS 1–2 n = 288	ICRS 3–4 N = 55	ICRS 1–4
<2 cm^2^	193	6	199
>2 cm^2^	95	49	144
*p*	0.001	0.02	

**Table 2:** Comparison of level of physical activity according to the size of articular cartilage defects in patellofemoral region.

ACD	PF < 2 cm^2^ n = 21	PF > 2 cm^2^ Nn = 12	*p*
<4 Tegner	95	116	0.04
>4 Tegner	104	28	0.03

## References

[1] Hinman R.S., Crossley K.M. (2007). Patellofemoral joint osteoarthritis: An important subgroup of knee osteoarthritis. Rheumatology.

[2] Cicuttini F., Ding C., Wluka A., Davis S., Ebeling P.R., Jones G. (2005). Association of cartilage defects with loss of knee cartilage in healthy, middle-age adults: A prospective study. Arthritis Rheum..

[3] Callaghan M.J., Guney H., Reeves N.D., Bailey D., Doslikova K., Maganaris C.N., Hodgson R., Felson D.T. (2016). A knee brace alters patella position in patellofemoral osteoarthritis: A study using weight bearing magnetic resonance imaging. Osteoarthritis Cartilage.

[4] Arendt E.A., Berruto M., Filardo G., Ronga M., Zaffagnini S., Farr J., Ferrua P., Grassi A., Condello V. (2016). Early osteoarthritis of the patellofemoral joint. Knee Surg. Sports Traumatol. Arthrosc..

[5] De Lange-Brokaar B.J., Bijsterbosch J., Kornaat P.R., Yusuf E., Ioan-Facsinay A., Zuurmond A.M., Kroon H.M., Meulenbelt I., Bloem J.L., Kloppenburg M. (2016). Radiographic progression of knee osteoarthritis is associated with MRI abnormalities in both the patellofemoral and tibiofemoral joint. Osteoarthritis Cartilage.

[6] Kim H.S., Yoo J.H., Park N.H., Chang J.H., Ban Y.S., Song S.H. (2016). Magnetic Resonance Imaging Findings in Small Patella Syndrome. Knee Surg. Relat. Res..

[7] Mehl J., Feucht M.J., Bode G., Dovi-Akue D., Sudkamp N.P., Niemeyer P. (2016). Association between patellar cartilage defects and patellofemoral geometry: A matched-pair MRI comparison of patients with and without isolated patellar cartilage defects. Knee Surg. Sports Traumatol. Arthrosc..

[8] Noehren B., Duncan S., Lattermann C. (2012). Radiographic parameters associated with lateral patella degeneration in young patients. Knee Surg. Sports Traumatol. Arthrosc..

[9] Aglietti P., Cerulli G. (1979). Chondromalacia and recurrent subluxation of the patella: A study of malalignment, with some indications for radiography. Ital. J. Orthop. Traumatol..

[10] Curl W.W., Krome J., Gordon E.S., Rushing J., Smith B.P., Poehling G.G. (1997). Cartilage injuries: A review of 31,516 knee arthroscopies. Arthroscopy.

[11] Hjelle K., Solheim E., Strand T., Muri R., Brittberg M. (2002). Articular cartilage defects in 1000 knee arthroscopies. Arthroscopy.

[12] Insall J., Falvo K.A., Wise D.W. (1976). Chondromalacia Patellae. A prospective study. J. Bone Joint Surg. Am. Vol..

[13] Indelicato P.A., Bittar E.S. (1985). A perspective of lesions associated with ACL insufficiency of the knee. A review of 100 cases. Clin. Orthop. Relat. Res..

[14] Lancourt J.E., Cristini J.A. (1975). Patella alta and patella infera. Their etiological role in patellar dislocation, chondromalacia, and apophysitis of the tibial tubercle. J. Bone Joint Surg. Am. Vol..

[15] Panni A.S., Cerciello S., Maffulli N., Di Cesare M., Servien E., Neyret P. (2011). Patellar shape can be a predisposing factor in patellar instability. Knee Surg. Sports Traumatol. Arthrosc..

[16] Aroen A., Loken S., Heir S., Alvik E., Ekeland A., Granlund O.G., Engebretsen L. (2004). Articular cartilage lesions in 993 consecutive knee arthroscopies. Am. J. Sports Med..

[17] Teichtahl A.J., Parkins K., Hanna F., Wluka A.E., Urquhart D.M., English D.R., Giles G.G., Cicuttini F.M. (2007). The relationship between the angle of the trochlear groove and patella cartilage and bone morphology—A cross-sectional study of healthy adults. Osteoarthritis Cartilage.

[18] Neusel E., Graf J. (1996). The influence of subchondral vascularisation on chondromalacia patellae. Arch. Orthop. Trauma Surg..

[19] Christoforakis J.J., Strachan R.K. (2005). Internal derangements of the knee associated with patellofemoral joint degeneration. Knee Surg. Sports Traumatol. Arthrosc..

[20] Tuna B.K., Semiz-Oysu A., Pekar B., Bukte Y., Hayirlioglu A. (2014). The association of patellofemoral joint morphology with chondromalacia patella: A quantitative MRI analysis. Clin. Imaging.

[21] Jungmann P.M., Tham S.C., Liebl H., Nevitt M.C., McCulloch C.E., Lynch J., Link T.M. (2013). Association of trochlear dysplasia with degenerative abnormalities in the knee: Data from the Osteoarthritis Initiative. Skeletal Radiol..

[22] Servien E., Ait Si Selmi T., Neyret P. (2003). Study of the patellar apex in objective patellar dislocation. Rev. Chir. Orthopedique Reparatrice Appar. Mot..

[23] Roos E.M., Engelhart L., Ranstam J., Anderson A.F., Irrgang J.J., Marx R.G., Tegner Y., Davis A.M. (2011). ICRS Recommendation Document: Patient-Reported Outcome Instruments for Use in Patients with Articular Cartilage Defects. Cartilage.

[24] Brattstrom H., Ahlgren S.A. (1959). Patellar shape and degenerative changes in the femoro-patellar joint. Acta Orthop. Scand..

[25] Kannus P., Niittymaki S. (1994). Which factors predict outcome in the nonoperative treatment of patellofemoral pain syndrome? A prospective follow-up study. Med. Sci. Sports Exerc..

